# The impact of smartphone addiction on depressive symptoms among medical students: a chain mediation model of self-acceptance and loneliness

**DOI:** 10.3389/fpsyg.2026.1773769

**Published:** 2026-04-09

**Authors:** Yuheng Wang, Ling Li, Xiaotian Yan

**Affiliations:** School of Psychology and Mental Health, North China University of Science and Technology, Tangshan, China

**Keywords:** depressive symptoms, loneliness, medical students, self-acceptance, serial mediation model, smartphone addiction

## Abstract

**Background:**

The primary importance of the mental health of medical students occurs in the context of the Healthy China 2030 Initiative since they will form the core workforce of the national medical and health system. Nevertheless, there is current data showing a surge in the prevalence of depressive symptoms in this group, with the overuse of smartphones cited as one of the key contributing factors.

**Objective:**

Although the strong relationship between smartphone addiction and depression has been established, there remain gaps in understanding the psychological processes that connect the two concepts. The sequential associations (i.e., cognitive and emotional processes) should be clarified, thus, it is imperative to at least identify the particular psychological pathway in the connection between smartphone addiction and depression in medical students.

**Methods:**

A cross-sectional survey was conducted on 823 medical students. To measure the levels of smartphone addiction, depressive symptoms, loneliness, and self-acceptance of participants, there was use of validated standardized questionnaires. The proposed Chain Mediation model was tested using SPSS 26.0.

**Results:**

Smartphone addiction is not only directly associated with depressive symptoms (β = 0.21, *p* < 0.001), but also shows an indirect association with them through three pathways. The chain mediation effect from “mobile addiction → self-acceptance → loneliness → depression” is significant (effect size = 0.18, 95% BootCI [0.09, 0.23]), while the independent mediation effect of self-acceptance is not significant.

**Conclusion:**

Smartphone addiction mainly may contribute to depressive symptoms in medical students in a dynamic chain reaction, that is, first, it is related to lower self-acceptance, which in turn is associated with higher levels of loneliness, and ultimately, linked to more severe depressive symptoms. The issue of interventions on the mental health of medical students cannot thus be limited to restrictions on smartphone use. Rather, there is a recommendation to employ strategy-based techniques aimed at enhancing self-acceptance and counteracting loneliness to be a more effective approach to breaking the smartphone addiction-depression chain.

## 1 Introduction

Healthy China 2030 Initiative has brought national mental health to a national strategic objective (Chen and Meier, 2021; Guo, 2016). This policy sets forth requirements for developing stronger and more standardized mental health service frameworks with special focus on early warning dynamics and supportive actions in key population groups, such as children and adolescents and working adults. It is on this policy background that a significant concern has been raised on the mental health of the emerging professional talents. The physical and mental health of medical students has a deep meaning as it is directly connected with the implementation of the “Healthy China 2030” Initiative and the quality and long-term progress of the healthcare sector in the country. However, there remains a critical challenge the unique characteristics of medical education (e.g., heavy academic workload, intensive clinical training) can have a considerable psychological burden on medical students. This pressure is substantially higher than it is in the case of non-medical students. The reasons for this pressure include a very heavy course load, high stress from clinical practice, and long-term exposure to job-specific stressors like seeing patient suffering and death. A large study that looked at data from around the world shows a worrying result: 27.2% of medical students have symptoms of depression.

A large number of them have serious psychological distress. This rate is much higher than in the general public or students in other majors (Hu et al., 2022; Li et al., 2022; Peng et al., 2023; Rotenstein et al., 2016). At the same time, technological advancement is accelerating rapidly. Information technology and mobile internet are now deeply part of medical education. Smartphones bring great convenience for studying, finding information, and accessing clinical data. But this technology has two sides. The bad side is that using smartphones too much, or being addicted to them, is becoming a new risky behavior. This might harm the mental health of medical students. Smartphone addiction, often termed problematic smartphone use, refers to a pattern of excessive and compulsive smartphone engagement that shares clinical features with behavioral addictions (e.g., loss of control, withdrawal, negative life consequences), although it is not formally classified as such in major diagnostic systems (e.g., DSM-5-TR, ICD-11). Its main signs are not being able to control smartphone use, needing to use it more and more, feeling bad when not using it, and it hurting your social life and schoolwork (James et al., 2023; Zhong et al., 2022). Against this backdrop, it is essential to conduct serious examination of psychological processes involved in association between depressive symptoms and smartphone addiction among medical students. This question is conciliated not only with national mental health strategies but also the desperate necessity of accurate and proactive mental healthcare services required in relation to this key population. This research will seek to develop and confirm a theoretical framework that explains the psychological processes underlying depression as a result of smartphone addiction in medical students. It is hoped that the findings will provide a solid theoretical basis for formulating specific prevention and interventional strategies and thus, have a significant theoretical and clinical implication.

### 1.1 The impact of smartphone addiction on depressive symptoms in medical students

A growing body of evidence has consistently shown the correlation between smartphone addiction and depressive symptoms. Such association is mainly supported by such theoretical frameworks as Compensatory Internet Use Theory (Liu et al., 2024; Hu et al., 2023; Montag et al., 2024) and cognitive-behavioral models (Qing et al., 2022; Schaffer et al., 2024). These theories suggest that persons tend to use digital technologies as maladaptive coping mechanism to avoid psychological pain. To medical students, who operate in highly-stressful environments, smartphones are a convenient means of escaping the burdens of academic pressure, difficult patient interactions, and career-related stress. This avoidance coping mechanism, however, causes a feedback loop of vicious cycles. To date, first, overuse of smartphones replaces the tasks that are crucial (including sleep, engagement with academic life, and face-to-face socialization), which increases susceptibility to academic burnout and sleep insufficiency, both well-known risk factors of depression (Liu et al., 2017; Krause et al., 2021). Second, the discontinuous and passive quality of most online communication harms learned concentration and critical reasoning, thereby undermining problem-solving capabilities and self-efficacy, which increases a sense of powerlessness. Large-scale meta-analyses of Chinese university students supports this relationship, with all studies showing that the degree of addiction to smartphones is significantly positively correlated with symptoms of depression (Yang et al., 2020). Similar outcomes are provided according to population-specific research on medical students: Huang et al. (2021), in particular, noted that the prevalence of smartphone addiction in the specified population was high and began to note that it is a direct predictor of the severity of symptoms of depression (Zhang et al., 2019). However, any type of simple correlation is not enough to construct mechanistic understanding (Hayes, 2017; Hayes and Rockwood, 2020). The development of theoretical frameworks and the design of evidence-based interventions require clarifying the psychological processes. Accordingly, the analysis of the intermediary characteristics of such variables as loneliness and self-acceptance is an essential milestone in the process of elucidating the mechanism between smartphone addiction and depression among medical students.

### 1.2 Relationship between smartphone addiction, loneliness, and depressive symptoms

Loneliness is that hurtful emotion that persists while believing the social relations are insufficient or poor in quantity. It is a significant interconnection between smartphone addiction and depression. To explain this, we can refer to two concepts: the Social Displacement Hypothesis and the unsuccessful placebo effect in the digital world. According to the Social Displacement Hypothesis, excessive time and efforts dedicated to smartphones and the internet may substitute in-person interpersonal communication. It implies that individuals are less likely and less willing to interact with others in real life, which undermines the existence of real relationship (Buecker et al., 2021; Chen et al., 2023; Kraut et al., 1998; Maples et al., 2024; Hascher and Waber, 2021). The students in medicine are high risk patients. Their work or school schedules are highly hectic and they have to switch, hence their social circles reduce. As they overly depend on socializing with their phones, they can be active online and alone offline. This further intensifies their loneliness (Jin and Park, 2013; Ponnusamy et al., 2020). Other students use their phones as they have the hope of finding a digital placebo effect. They believe that communicating through their phones would substitute in-person interactions, and they will feel less isolated. However, numerous studies indicate that basic internet behaviors, such as going through the feed or commenting likes, do not provide actual emotional reinforcement and identification. Actually, they may even exacerbate loneliness. It occurs when individuals contrast the idealized happy lives of others with the lives that they live (Singh et al., 2022; Keyes and Platt, 2024; Verduyn et al., 2017). So, there is a gap between what they hope for and what actually happens. This can turn the smartphone from a possible solution into something that makes the problem worse. Also, loneliness itself is more than just a bad feeling. According to the evolutionary model by Cacioppo et al. (2006), it triggers negative thought patterns and physical stress responses. This greatly increase a person’s risk of developing depression (Cacioppo et al., 2006; Kniffin et al., 2021; Vahedi and Saiphoo, 2018; Wang et al., 2018). Real-world studies support this path. For example, long-term studies have found that problematic smartphone use leads to later depressive symptoms by first increasing loneliness in university students (Elhai et al., 2016; Pan et al., 2023; Kupferberg and Hasler, 2023; Liu and Wang, 2011). Because of this, we propose that for medical students, smartphone addiction may be linked to more severe depressive symptoms via its association with increased feelings of loneliness.

### 1.3 The serial mediating role of self-acceptance and loneliness

We have looked at loneliness and self-acceptance separately. Now, a bigger question is: do they work on their own, or do they connect in a sequence, creating a chain from smartphone addiction to depression? Evidence suggests they are connected in a sequence. This means loneliness and self-acceptance likely work together in a serial pathway. According to psychological theories, individuals form our sense of self according to our relationship with other individuals (Brown, 2022; Cooley, 2017). Sociometer theory further posits that human beings have the fundamental need of belonging. To positively support ourselves, we need a feeling of connection with others (Barrett et al., 2019; Gilbert et al., 1998). To a medical student, an addiction to smartphone may create feelings of loneliness and antisocial behavior. This implies that they are less likely to receive positive feedbacks, emotional care, and validation among others. It becomes more difficult to establish a positive and strong self-image without this social confirmation. This renders the challenge of accepting them as they are, and their shortcomings (Li et al., 2020; Huang and Zhao, 2020; Mellor et al., 2008; Zhang and Li, 2020). As an example, a student sitting alone may reason out and say, I am isolated since I am not likeable. This reflection reduces their self-acceptance. In perspective, a low self-acceptance individual does not like himself/herself. In many cases, they do not have confidence in social circumstances and are afraid of rejection. Due to this they might be afraid of socializing. Their social isolation and loneliness is further intensified by this avoidance (Barrett et al., 2019; Cacioppo et al., 2006). This can create a cycle where loneliness and low self-acceptance reinforce each other. Therefore, we propose a specific sequence for medical students. First, smartphone addiction increases feelings of loneliness (an emotional effect). This loneliness then damages their self-acceptance (a thinking effect). Finally, this combination of bad feelings and negative thoughts leads to more severe depressive symptoms. Previous studies have looked at loneliness or self-acceptance alone. But putting them together in a chain, especially for medical students’ smartphone use and depression, is a new area. Studying this will help us gain a comprehensive understanding of how smartphone addiction harms mental health by showing the connection between emotional and thinking processes.

### 1.4 Research hypotheses

**Figure 1:**
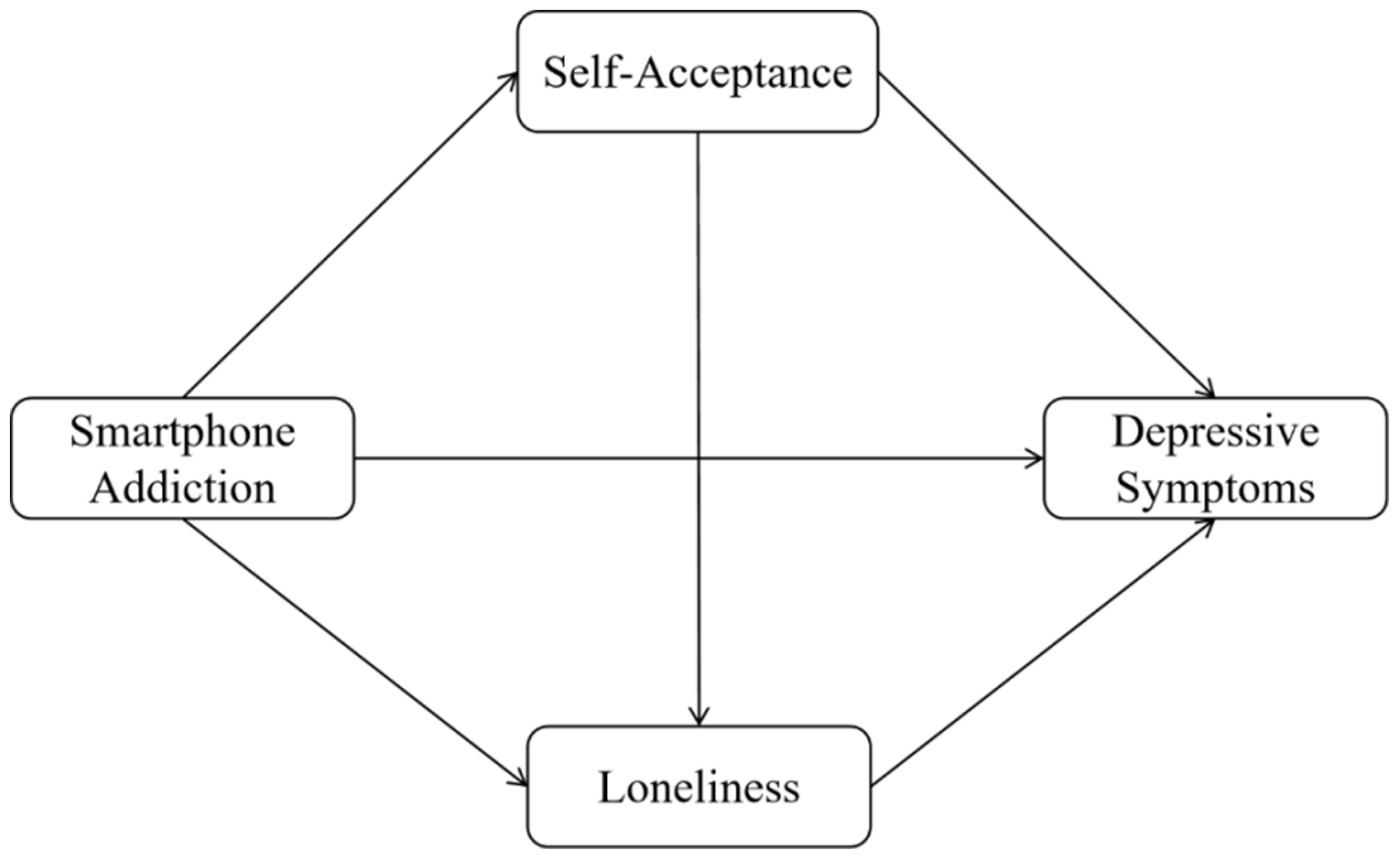
A chain mediation model of self-acceptance and loneliness.

Based on the systematic theoretical reasoning and literature review above, this study constructs a Chain Mediation model (see Figure 1) to thoroughly investigate the psychological mechanisms by which smartphone addiction affects depressive symptoms in medical students, focusing specifically on the independent and serial mediating roles of loneliness (affective pathway) and self-acceptance (cognitive pathway). The following hypotheses are proposed:

*H1*: Smartphone addiction will be significantly and positively associated with the level of depressive symptoms.

*H2*: Loneliness will independently mediate the relationship between smartphone addiction and depressive symptoms. Specifically, higher levels of smartphone addiction will be associated with greater loneliness, which in turn will be associated with more severe depressive symptoms.

*H3*: Self-acceptance will independently mediate the relationship between smartphone addiction and depressive symptoms. Specifically, higher levels of smartphone addiction will be associated with lower self-acceptance, which in turn will be associated with more severe depressive symptoms.

*H4*: Loneliness and self-acceptance will serially mediate the relationship between smartphone addiction and depressive symptoms. Specifically, smartphone addiction is positively associated with loneliness (affective pathway), which is negatively associated with self-acceptance (cognitive pathway), and this sequential pathway is linked to more severe depressive symptoms.

## 2 Methods

### 2.1 Participants and procedure

A cross-sectional survey was conducted between September and November 2023. Utilizing a cluster sampling method, a total of 850 full-time medical students across all five academic years (Years 1 to 5) were recruited from the medical school of a comprehensive university in China. The sample encompassed various majors, including Clinical Medicine, Anesthesiology, Medical Imaging, and Preventive Medicine. After obtaining approval from relevant university departments and class advisors, and securing informed consent from all participants, questionnaires were administered by trained researchers during class intervals following standardized instructions.

Of the 850 questionnaires distributed, after excluding questionnaires with patterned responses (e.g., straight-line answering), excessively short completion times, or missing key data, 823 valid questionnaires were retained for final analysis, resulting in a valid response rate of 96.8%. The final sample consisted of 419 males (50.9%) and 404 females (49.1%), aged between 17 and 25 years (*M* = 20.24, SD = 1.63). The distribution across academic years was as follows: Year 1 (22.0%, *n* = 173), Year 2 (21.6%, *n* = 170), Year 3 (20.5%, *n* = 161), Year 4 (18.6%, *n* = 146), and Year 5 (17.3%, *n* = 136). Independent samples *t*-tests and chi-square tests revealed no significant differences (*p* > 0.05) on key demographic variables between participants who were excluded and those included in the final analysis, suggesting that participant attrition did not significantly bias the sample. The study protocol was approved by the University Ethics Review Board (2024YFC2707800).

### 2.2 Measures

#### 2.2.1 Smartphone addiction

The Smartphone Addiction Inventory (SPAI), developed by Leung (2008) and widely used in Chinese populations, was adopted to assess symptoms of smartphone addiction. The 17-item scale measures four dimensions: withdrawal symptoms (e.g., “I feel uneasy if I don’t have my smartphone with me”), loss of control (e.g., “I find myself using my smartphone longer than I intended”), escape (e.g., “I use my smartphone to relax when I feel lonely”), and inefficiency (e.g., “My academic performance has declined due to excessive smartphone use”). Items are rated on a 5-point Likert scale from 1 (never) to 5 (always), with higher total scores (range 17–85) indicating greater severity of smartphone addiction. The SPAI has demonstrated good reliability and validity in Chinese contexts. In this study, confirmatory factor analysis (CFA) supported the four-factor structure: *χ*^2^*/df* = 2.85, *CFI* = 0.94, *TLI* = 0.92, *RMSEA* = 0.065. The scale also showed excellent internal consistency, with a Cronbach’s α of 0.93 for the total score.

#### 2.2.2 Depressive symptoms

The Center for Epidemiologic Studies Depression Scale (CES-D; Radloff, 1977), a widely used self-report measure, was used to assess the frequency of depressive symptoms over the past week. The 20-item scale captures six core aspects of depressive affect: depressed mood, feelings of guilt and worthlessness, feelings of helplessness and hopelessness, psychomotor retardation, loss of appetite, and sleep disturbances. Items are rated on a 4-point scale from 0 (rarely or none of the time [<1 day]) to 3 (most or all of the time [5–7 days]), with total scores ranging from 0 to 60. Higher scores indicate more severe depressive symptoms, with a commonly used clinical cutoff score of 16. CFA confirmed the scale’s structure in our sample: *χ*^2^*/df* = 3.12, *CFI* = 0.93, *TLI* = 0.91, *RMSEA* = 0.068. The CES-D demonstrated excellent internal consistency (α = 0.92).

#### 2.2.3 Loneliness

The UCLA Loneliness Scale (Version 3; Russell, 1996), a gold-standard measure, was used to evaluate subjective feelings of loneliness. The scale’s 20 items directly describe experiences of loneliness (e.g., “I lack companionship”) rather than its potential causes. Eleven items are positively worded and nine are reverse-scored (e.g., “I feel part of a group of friends”). Responses are given on a 4-point Likert scale from 1 (never) to 4 (often), with total scores ranging from 20 to 80; higher scores reflect greater loneliness. CFA supported a unidimensional structure: *χ*^2^*/df* = 2.98, *CFI* = 0.95, *TLI* = 0.94, *RMSEA* = 0.063. The scale showed high internal consistency (α = 0.90).

#### 2.2.4 Self-acceptance

The Self-Acceptance Questionnaire (SAQ), developed by Cong and Gao (1999) based on humanistic psychology theory, was used to assess self-acceptance. This 16-item instrument comprises two dimensions: self-evaluation (8 items assessing cognitive appraisal of the self, e.g., “I hold a positive attitude toward myself”) and self-acceptance (8 items assessing affective acceptance of one’s strengths and weaknesses, e.g., “I feel uneasy about my shortcomings” [reverse-scored]). Items are rated on a 4-point Likert scale from 1 (strongly disagree) to 4 (strongly agree). Total scores range from 16 to 64, with higher scores indicating greater self-acceptance. CFA confirmed the two-factor structure: *χ*^2^*/df* = 3.20, *CFI* = 0.92, *TLI* = 0.90, *RMSEA* = 0.070. The SAQ demonstrated good reliability, with a total scale α of 0.88, and subscale α coefficients of 0.84 (self-evaluation) and 0.93 (self-acceptance).

### 2.3 Data analysis

Data were analyzed using SPSS 26.0. First, descriptive statistics and Pearson correlations were calculated, and Harman’s single-factor test was used to check for common method bias. Next, proposed model was tested. This model looked at the chain of effects from smartphone addiction to depressive symptoms through loneliness and then self-acceptance, using Model 6 from the SPSS PROCESS macro (Version 4.1 by Hayes) for this test. To determine the significance of indirect effects, bootstrap confidence intervals were used with 5,000 resamples. An effect is considered significant if its 95% bias-corrected bootstrap confidence interval does not include zero. Harman’s single-factor test revealed that the first factor accounted for 23.855% of the total variance, which is below the 50% threshold, suggesting that common method bias was not a major concern.

## 3 Results

### 3.1 Preliminary analyses

First, we conducted a preliminary screening using the Harman single-factor test, which revealed that the first non-rotated factor accounted for 23.86% of the total variance, which was not statistically significant. Given the limitations of this method, we adopted a more robust statistical control approach to further rigorously examine common method bias. Within the framework of confirmatory factor analysis, we constructed a model incorporating a latent method factor, loading all items from each scale to estimate the magnitude of methodological variation. This model was then compared with the benchmark four-factor measurement model. The results demonstrated that the inclusion of the method factor did not significantly improve the model fit indices (*ΔCFI* < 0.01, *ΔTLI* < 0.01, *ΔRMSEA* < 0.015), and the proportion of variance explained by the method factor remained limited. These findings suggest that while the influence of common method bias cannot be entirely ruled out, its impact on the estimation of key inter-variable associations in this study is unlikely to pose a serious threat.

Descriptive statistics and intercorrelations for all study variables are presented in Table 1. As hypothesized, smartphone addiction was positively correlated with depressive symptoms (*r* = 0.23, *p* < 0.01) and negatively correlated with self-acceptance (*r* = −0.69, *p* < 0.01). Self-acceptance was negatively correlated with depressive symptoms (*r* = −0.28, *p* < 0.01). Loneliness was positively correlated with depressive symptoms (*r* = 0.52, *p* < 0.01) and negatively correlated with self-acceptance (*r* = −0.33, *p* < 0.01). A small but significant positive correlation was found between smartphone addiction and loneliness (*r* = 0.09, *p* < 0.05). These correlations provided preliminary support for testing the mediation hypotheses.

**Table 1:** Descriptive statistics and correlations for study variables (*N* = 823).

Variable	*M*	*SD*	1	2	3	4
1. Smartphone addiction	2.53	0.81	–			
2. Self-acceptance	2.11	0.63	−0.69**	–		
3. Loneliness	2.48	0.30	0.09*	−0.33**	–	
4. Depressive symptoms	2.26	0.46	0.23**	−0.28**	0.52**	–

*p < 0.05, **p < 0.01.

### 3.2 Testing the chain mediation model

The results of the Chain Mediation analysis are summarized in Table 2. In the first equation, smartphone addiction significantly and negatively associated with self-acceptance (β = −0.69, *t* = −26.92, *p* < 0.001). In the second equation, after controlling for smartphone addiction, self-acceptance significantly and negatively associated with loneliness (β = −0.51, *t* = −11.52, *p* < 0.001). Smartphone addiction also had a significant direct negative effect on loneliness (β = −0.26, *t* = −5.92, *p* < 0.001). In the final equation predicting depressive symptoms, when smartphone addiction, self-acceptance, and loneliness were included simultaneously, only loneliness emerged as a significant positive predictor (β = 0.51, *t* = 16.11, *p* < 0.001). The direct path from self-acceptance to depressive symptoms was not significant (β = 0.03, *t* = 0.79, *p* = 0.433). The direct effect of smartphone addiction on depressive symptoms remained significant (β = 0.21, *t* = 5.12, *p* < 0.001), as did the total effect (β = 0.23, *t* = 6.81, *p* < 0.001).

**Table 2:** Chain mediation model testing the effects of smartphone addiction on depressive symptoms (*N* = 823).

Equation	Outcome	Predictor	β	*t*	*p*
1	Self-acceptance	Smartphone addiction	−0.69	−26.92	< 0.001
2	Loneliness	Smartphone addiction	**−0.26**	**−5.92**	**< 0.001**
		Self-acceptance	**−0.51**	**−11.52**	**< 0.001**
3	Depressive symptoms	Smartphone addiction	**0.21**	**5.12**	**< 0.001**
		Self-acceptance	**0.03**	**0.79**	**0.433**
		Loneliness	**0.51**	**16.11**	**< 0.001**
Total effect	Depressive symptoms	Smartphone addiction	0.23	6.81	< 0.001

**Figure 2:**
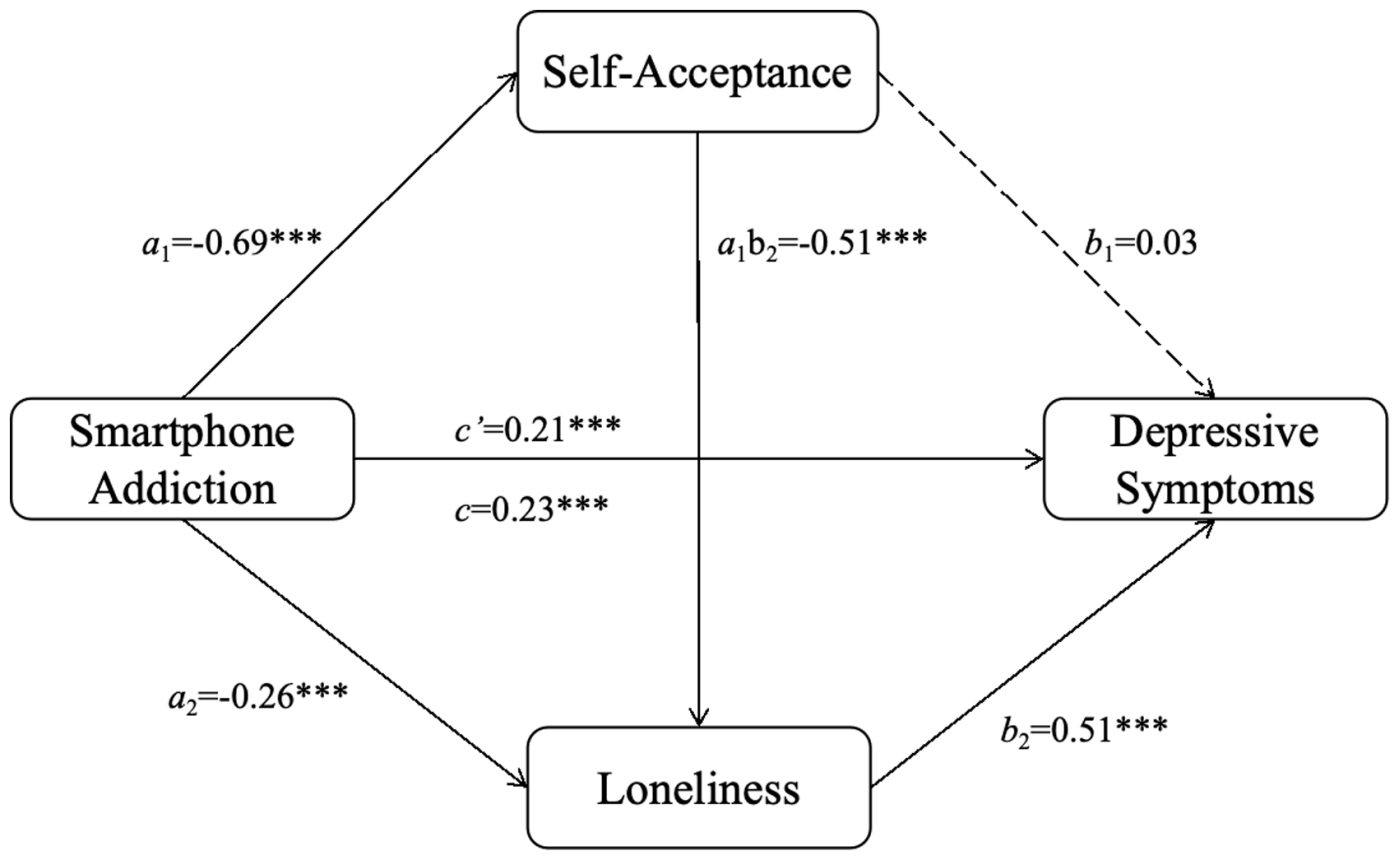
Testing the chain mediation model. ***p < 0.05.

Bootstrap analysis for the indirect effects (see Table 3) provided support for the hypothesized Chain Mediation (see Figure 2). The specific indirect effect for the path Smartphone Addiction → Self-Acceptance → Loneliness → Depressive Symptoms was significant (effect = 0.18, 95% BootCI [0.09, 0.23]). The indirect effect for the path Smartphone Addiction → Loneliness → Depressive Symptoms was also significant (effect = −0.13, 95% BootCI [−0.18, −0.04]). In contrast, the indirect effect for the path Smartphone Addiction → Self-Acceptance → Depressive Symptoms was not significant (effect = −0.02, 95% BootCI [−0.07, 0.05]). The total indirect effect was not significant (effect = 0.02, 95% BootCI [−0.04, 0.12]). The direct effect (effect = 0.21, 95% BootCI [0.13, 0.29]) and total effect (effect = 0.23, 95% BootCI [0.17, 0.30]) of smartphone addiction on depressive symptoms were both significant. These results indicate that self-acceptance and loneliness primarily function as serial mediators rather than parallel mediators in the relationship between smartphone addiction and depressive symptoms.

**Table 3:** Bootstrap analysis for indirect effects (*N* = 823).

Path	Effect	BootSE	95% Boot LLCI	95% Boot ULCI
Total indirect effect	0.02	0.04	−0.04	0.12
Ind1: SA → Self-acceptance → DS	−0.02	0.03	−0.07	0.05
Ind2: SA → Loneliness → DS	−0.13	0.04	−0.18	−0.04
Ind3: SA → Self-acceptance → Loneliness → DS	0.18	0.04	0.09	0.23
Total effect	0.23	0.03	0.17	0.30
Direct effect	0.21	0.04	0.13	0.29

Notably, the indirect effect for the path “smartphone addiction → loneliness → depressive symptoms” was negative, which may suggest a suppression effect (effect = −0.013, 95% BootCI [−0.18, −0.04]). This indicates that when self-acceptance is controlled, the direct relationship between smartphone addiction and loneliness reverses direction, underscoring the complex interplay among these variables rather than a simple mediation.

## 4 Discussion

Based on the Compensatory Internet Use Theory and cognitive-behavioral models, this study constructed a chain mediation model to deeply explore the intrinsic psychological mechanisms through which smartphone addiction affects depressive symptoms in medical students. By analyzing cross-sectional survey data from 823 medical students, the main findings were: (1) Smartphone addiction was significantly and positively associated with depressive symptoms, consistent with hypothesis H1; (2) Correlation analyses showed smartphone addiction was significantly positively correlated with loneliness and depression, and significantly negatively correlated with self-acceptance, while self-acceptance was significantly negatively correlated with both loneliness and depression, these results provide a basis for mediation effect testing; (3) The chain mediation model analysis indicated that loneliness and self-acceptance played important mediating roles. Specifically, smartphone addiction influenced depression not merely through independent parallel pathways, but primarily through the sequential chain pathway: “smartphone addiction → self-acceptance → loneliness → depression”. That is, higher levels of smartphone addiction were linked to lower self-acceptance among medical students, which was in turn associated with higher levels of loneliness, collectively correlating with an increased risk of depressive symptoms. However, the independent mediating effect of self-acceptance was not supported, and the independent mediating effect of loneliness, while significant, had a negative effect size, opposite in direction to the direct effect, suggesting complexity in the relationship. The chain mediation model represents a core statistical pathway linking these variables. The following sections discuss these findings in depth in relation to existing literature.

### 4.1 The direct effect of smartphone addiction on depressive symptoms in medical students

This study found that Smartphone addiction was significantly and positively associated with depressive symptoms in medical students, a result highly consistent with numerous existing studies. Firstly, considering the specific characteristics of the population, medical students, as the future backbone of the healthcare system, face long-term excessive academic pressure, high professional standards, and emotional exhaustion in clinical practice, making their mental health a subject of concern. Many large reviews of studies show that medical students have much higher rates of depressive symptoms than the general public or students in other fields (Hu et al., 2022; Li et al., 2022; Rotenstein et al., 2016; Peng et al., 2023). In this situation, smartphones are very common and useful. But excessive use or addictive use can exacerbate psychological risks. According to the Compensatory Internet Use Theory, people might use the internet and smartphones to escape from real-life stress and bad feelings (Liu et al., 2024; Hu et al., 2023). For medical students, smartphones can easily become a “digital escape.” However, this way of coping by avoiding problems often has the opposite effect. Spending too much time online takes away time needed for rest, studying, and real-life social activities, which in turn leads to issues such as academic burnout and not getting enough sleep, which are known to cause depression (Yang et al., 2020; Alonzo et al., 2021). The results of this study, along with other research on Chinese medical students (Huang, 2022; Zhang et al., 2019), confirm a consistent conclusion: smartphone addiction is a strong and reliable predictor of depressive symptoms in this group. This means that paying attention to how medical students use their smartphones is very important. It can help identify depression early and provide targeted support.

### 4.2 The mediating role of loneliness

This study confirmed the key mediating role of loneliness in the relationship between smartphone addiction and depression, but the specific pathway showed complexity. Bootstrap test results indicated that the indirect effect for the path “smartphone addiction → loneliness → depression” was significant, but the effect size was negative. This appears superficially inconsistent with theoretical expectations and simple correlation analysis (which showed a weak positive correlation between smartphone addiction and loneliness). This result might indicate that, after controlling for other variables (particularly self-acceptance), the direct effect of smartphone addiction on loneliness changed direction, or a suppression effect exists. However, more importantly, loneliness, as a core element of the affective pathway, played a crucial role in the chain mediation model (smartphone addiction → self-acceptance → loneliness → depression), with a significant effect size. This finding can be explained by two theories: the “social displacement hypothesis” and social comparison theory. The social displacement hypothesis posits that spending too much time on smartphones replaces face-to-face social interaction. This causes real-world social relationships to become more fragile (Kraut et al., 1998; Buecker et al., 2021). Medical students already have busy schedules that make social life hard. If they rely too much on online socializing through their phones, they can become “active online but withdrawn offline.” This leads to stronger feelings of loneliness (Jin and Park, 2013). Also, using social media often involves comparing yourself to others who seem to be doing better. When medical students see posts about other people’s seemingly more successful and happier lives, it can easily make them feel inadequate and isolated (Liu et al., 2017; Verduyn et al., 2017). According to the evolutionary theory model by Cacioppo et al. (2006), loneliness is not just a painful feeling. It also triggers negative thought patterns, like being overly alert to social threats, and stress responses. This greatly increases the risk of depression. The results of our study support this chain of events. They show that smartphone addiction makes depressive symptoms worse in medical students by first increasing loneliness. So, loneliness acts as an important emotional bridge. It connects the external behavior (smartphone addiction) to the internal emotional problem (depression).

### 4.3 The mediating role of self-acceptance

This study found that self-acceptance did not have a significant mediating effect on its own. However, its role at the start of the chain was very important. Smartphone addiction strongly and negatively predicted self-acceptance. This means that addictive smartphone use seriously harms a person’s self-awareness and self-acceptance. From a cognitive-behavioral perspective, smartphone addiction can be seen as a negative “self-related event.” When medical students realize they are spending too much time on unproductive phone activities, instead of studying or improving, it can easily cause feelings of self-blame, regret, and a feeling of being out of control. If not effectively managed, this dissatisfaction with one’s own behavior can generalize into overall negative evaluations of one’s abilities and willpower (e.g., “I can’t even control my phone use, I’m a failure”), significantly lowering self-acceptance (Qin et al., 2020). Individuals with low self-acceptance are typically harshly self-critical, struggle to tolerate imperfection, and are more prone to intense feelings of incompetence and worthlessness when facing academic setbacks or interpersonal difficulties – core components of the cognitive triad of depression (Beck et al., 1996). Although in the full model of this study, the direct predictive effect of self-acceptance on depression was not significant (potentially masked by loneliness), this precisely suggests that the influence of self-acceptance might primarily be realized through subsequent affective experiences like loneliness. Ryff and Keyes (1995) consider self-acceptance a core dimension of psychological well-being, and high self-acceptance is an important psychological resource for coping with stress (MacInnes, 2006; Kupferberg and Hasler, 2023). Therefore, smartphone addiction lays the groundwork for depression by weakening this key psychological resource.

### 4.4 The chain mediating role of self-acceptance and loneliness

The most significant finding of this study is the identification of a significant chain mediation effect of self-acceptance and loneliness between smartphone addiction and depressive symptoms in medical students, specifically the path “smartphone addiction → self-acceptance → loneliness → depression”. This chain effect (effect size 0.18) was substantially larger than the effects of other paths, indicating that the impact of smartphone addiction on depression primarily occurs through a sequential, progressive internal process: first damaging self-cognition (reducing self-acceptance), which then triggers negative socio-affective experiences (increasing loneliness). This finding effectively integrates cognitive and affective pathways, supporting social constructivist theories of self-concept. According to Cooley’s (2017) “looking-glass self” theory and sociometer theory, an individual’s self-concept is largely formed through social interactions, by perceiving others’ evaluations and reactions (Brown, 2022; Gilbert et al., 1998). For medical students, low self-acceptance caused by smartphone addiction can undermine their social confidence. When an individual holds negative perceptions and beliefs about themselves, he or she might be nervous in a social context. This shyness of social situations, probably caused by fear of verbal disorder, may aggravate the state of loneliness and strengthen the detachment (Cacioppo et al., 2006). At the same time, there is the potential to develop a sense of isolation even in the online space because of compulsive smartphone use. This dual dynamic restricts access to positive social feedback and support which are essential to the formation of a coherent and stable self-concept. The ability to self-accept, therefore, can be compromised (Barrett et al., 2019). A cyclical tendency can therefore arise where low levels of self acceptance lead to further increase in loneliness and even low levels of loneliness lead to low levels of self acceptance. This complete mediation model is the first to be empirically tested in the current study, involving the relationship between smartphone addiction and depression. The results reveal that the addiction to smartphones initially disrupts medical students’ self-expectation. This damaged self-perception in turn impacts negatively on their social and emotional functioning, and the psychological disturbances together lead to the depressive symptoms in the end. This finding explains the intrapersonal channel in which addiction to smartphones affects mental health, whereby a complicated interaction between cognition and emotions occurs.

This study employed the CES-D scale to assess depressive symptoms, whose high validity and broad applicability have been supported by extensive research. However, caution should be exercised when interpreting CES-D scores in the specific population of medical students. The CES-D includes a significant proportion of somatization and anhedonia items (e.g., “restless sleep,” “feeling tired,” “loss of appetite”). Medical students generally face high academic pressure, irregular schedules, and physical exhaustion from clinical rotations, which can themselves lead to similar somatic symptoms. Additionally, excessive smartphone use has been demonstrated to directly interfere with sleep quality and increase fatigue. Therefore, the observed depressive symptom scores in this study may, to some extent, reflect non-specific psychological distress related to situational stress and behavioral consequences rather than a strict, clinically diagnostic depressive syndrome. This overlap in symptom sources may influence the absolute prevalence estimation of depressive symptoms and potentially amplify the strength of the direct association between smartphone addiction and depressive symptoms. Nevertheless, the primary contribution of this study lies in revealing the association patterns and psychological mechanism pathways among variables. The core of the chain mediation model (smartphone addiction → self-acceptance → loneliness → depressive symptoms) we validated lies in the sequential effects of cognitive self-evaluation (self-acceptance) and emotional experience (loneliness). The theoretical construction of this pathway is based on psychosocial processes, and its validity is relatively less influenced by specific somatic symptom sources. In other words, even if some variance in depressive symptoms stems from stress or behavioral consequences, the association pathways revealed by this study model—mediated through self-awareness and socio-emotional experiences—still provide a valuable mechanistic perspective for understanding the complex link between smartphone-related behaviors and overall psychological distress. Future research could further isolate and clarify these effects by using depression measurement tools that focus more on cognitive-emotional core symptoms, or by controlling for covariates such as academic stress and objective sleep indicators.

## 5 Limitations and future directions

There are a number of limitations that this research has brought about and it is important to note them in order to address them in future research. To begin with the research design, the study adopted a cross-sectional research design. Such a methodology only describes the concurrent correlations between variables but not the causality. The use of longitudinal tracking or experimental designs should be embraced in future research to establish the dynamics over time and the possible causal mechanisms between the constructs being tested. Second, data were all based on self-reports of the participants. Although validity tests did not show that this bias was extremely large, self-report measures are always vulnerable to social desirability response and recall errors. Future studies can be able to incorporate more objective data, like objective screen time measurement, peer ratings or assessments by clinicians, to triangulate the data and increase reliability. Third, the researchers narrowed down the sample of medical students in one region of China, which can probably restrict the external validity of the results. These outcomes should be cautiously applied in the process of generalizing these findings to other cultural settings, non-medical groups of students, or the general population. Further research should include sample of different schools, different regions, or cross-national cohorts. Fourth, although we implemented procedural and statistical controls (e.g., anonymization, item order balance, and latent methodological factor testing) in our study design and statistical analysis, all data were derived from student self-reports, which still poses a risk of common method bias. Future research could incorporate multi-source data (e.g., peer evaluations, objective mobile phone usage duration) or adopt longitudinal designs to better distinguish between methodological variations and the true relationship between constructs. Fifth, regarding measurement, the somatic symptoms assessed by the CES-D scale in this study may overlap with the behavioral consequences of high stress, irregular sleep patterns, and smartphone overuse commonly experienced by medical students. While this does not negate the theoretical value of the variable association patterns and mediating pathways revealed in this study, future research should employ more refined measurement strategies (e.g., distinguishing core affective symptoms from somatic symptoms, or controlling for relevant covariates) to more accurately estimate specific effect sizes.

Additionally, the cultural and educational context of Chinese medical students may shape the observed relationships. Within China’s highly competitive medical education system, students often face intense academic pressure and socialization patterns that may amplify the role of smartphone use as both a coping tool and a source of emotional isolation. Collectivist cultural norms could also influence expressions of loneliness and self-acceptance. Future cross-cultural studies are encouraged to examine the generalizability of this sequential pathway.

### 5.1 Theoretical and practical implications

Despite these weaknesses, this research is valuable to both theory and practice. It combines the most important theoretical constructs, including the Compensatory Internet Use Theory and the theory of self-concept, and empirically validates a model which describes a pathway between smartphone addiction and depressive symptoms on an empirical basis. This goes beyond making associations to making suggestions of a plausible mechanism in psychology. Moreover, the study bridges the gap between two constructs that are frequently investigated separately, self-acceptance (cognitive-evaluative) and loneliness (affective experience). It is also interesting to note that the researchers concluded that low self-acceptance is an aggravator of loneliness, which adds depth to our understanding of how behavioral problems translate into emotional distress. The practical implications of the findings to the support of the mental well-being of medical students are also obtained. (1) Universities are challenged to keep track of smartphone clocks and start considering the incorporation of mental health education on digital wellness into the existing mental health programs. (2) Interventions must not only focus on limiting time of screen time; the root cause of psychological vulnerabilities should be considered. The cognitive-behavioral therapy (CBT) or acceptance and commitment therapy (ACT) may be used as modalities that would make people have a positive self-image and acceptance, thus, decreasing self-criticism and improving psychological resilience. (3) Social connectedness: The institutions are to ensure that social connectedness is supported through the promotion of study groups, interests-related clubs, or peer-support networks. This type of initiatives might aid students in gaining significant connections and alleviating their sense of loneliness. The interventions that address both self-acceptance and social connectedness can be the most effective to interfere with the depression pathway caused by the addictive behavior. Overall, the work gives clear empirical grounds on how to create dedicated assistance to this risky population. The fact that it aligns with the aims of the Healthy China 2030 initiative highlights its relevance to the improvement of mental health services.

## 6 Conclusion

This paper constructed and established a model to explain how smartphone addiction contributes to depression among medical students. The major result revealed an indirect association between smartphone addiction and depression through a specific sequential pathway: self-acceptance, loneliness. In the proposed model, smartphone overuse is associated with lower self-acceptance of individuals initially; the compromized self-acceptance, in its turn, increases the feelings of loneliness, which further aggravates depressive symptoms. The conclusion explains the association between addictive behavior and depressive emotion as critical roles of intrapersonal (self-perception) and interpersonal experiences (loneliness). Therefore, the issue of the mental health of medical students will also require more than superficial rules on screen time- it will require a full-fledged intervention system. This model must introduce evidence-based interventions like Cognitive Behavioral Therapy (CBT) to increase self-acceptance, and at the same time promote the formation of face to face social interactions to reduce loneliness. These two measures can be applied synergistically to disrupt the psychological process of smartphone addiction to depression, which can offer valuable lessons in how medical experts can be trained in a manner that allows them to be competent and mentally sound.

## Data Availability

The original contributions presented in the study are included in the article/supplementary material, further inquiries can be directed to the corresponding author.
